# Analysis of transgenic zebrafish expressing the Lenz-Majewski syndrome gene *PTDSS1* in skeletal cell lineages

**DOI:** 10.12688/f1000research.17314.1

**Published:** 2019-03-11

**Authors:** Marian Seda, Emma Peskett, Charalambos Demetriou, Dale Bryant, Gudrun E. Moore, Philip Stanier, Dagan Jenkins

**Affiliations:** 1 GOS Institute of Child Health, University College London, London, WC1N 1EH, UK

**Keywords:** Lenz-Majewski syndrome, Tol2-kit

## Abstract

**Background:** Lenz-Majewski syndrome (LMS) is characterized by osteosclerosis and hyperostosis of skull, vertebrae and tubular bones as well as craniofacial, dental, cutaneous, and digit abnormalities. We previously found that LMS is caused by *de novo* dominant missense mutations in the *PTDSS1* gene, which encodes phosphatidylserine synthase 1 (PSS1), an enzyme that catalyses the conversion of phosphatidylcholine to phosphatidylserine. The mutations causing LMS result in a gain-of-function, leading to increased enzyme activity and blocking end-product inhibition of PSS1.

**Methods:** Here, we have used transpose-mediated transgenesis to attempt to stably express wild-type and mutant forms of human *PTDSS1 *ubiquitously or specifically in chondrocytes, osteoblasts or osteoclasts in zebrafish.

**Results:** We report multiple genomic integration sites for each of 8 different transgenes. While we confirmed that the ubiquitously driven transgene constructs were functional in terms of driving gene expression following transient transfection in HeLa cells, and that all lines exhibited expression of a heart-specific cistron within the transgene, we failed to detect *PTDSS1 *gene expression at either the RNA or protein levels in zebrafish. All wild-type and mutant transgenic lines of zebrafish exhibited mild scoliosis with variable incomplete penetrance which was never observed in non-transgenic animals.

**Conclusions:** Collectively the data suggest that the transgenes are silenced, that animals with integrations that escape silencing are not viable, or that other technical factors prevent transgene expression. In conclusion, the incomplete penetrance of the phenotype and the lack of a matched transgenic control model precludes further meaningful investigations of these transgenic lines.

## Introduction

Lenz-Majewski syndrome (LMS; MIM 151050) is a rare disease, characterized by a complex set of clinical features that are progressive and potentially life limiting. These include osteosclerosis and hyperostosis of skull, vertebrae and tubular bones as well as craniofacial, dental, cutaneous, and digit abnormalities. The patients also have severe growth restriction and moderate to severe intellectual disability ( Sousa *et al*., 2014). To date, only 16 affected individuals have been reported worldwide ( Chrzanowska *et al*., 1989; Dateki *et al*., 2007; Gorlin & Whitey, 1983; Mizuguchi *et al*., 2015; Nishimura *et al*., 1997; Piard *et al*., 2018; Saraiva, 2000; Shoja *et al*., 2013; Sousa *et al*., 2014; Tamhankar *et al*., 2015; Wattanasirichaigoon *et al*., 2004; Whyte *et al*., 2015). We previously found that LMS is caused by *de novo* dominant missense mutations in the *PTDSS1* gene ( Sousa *et al*., 2014). *PTDSS1* encodes an enzyme, phosphatidylserine synthase 1 (PSS1), which catalyses the base-exchange reaction of serine for choline such that phosphatidylcholine is converted to phosphatidylserine (PS). PSS1 is in fact one of two enzymes (along with PSS2) involved in the production of PS, where in an alternative pathway, PSS2 catalyses a similar serine exchange reaction with phosphatidylethanolamine. PS is a quantitatively minor, but physiologically important phospholipid present in all mammalian cells (Vance *et al*., 2018). In addition to its role as a constituent of membranes, PS is also known to play important roles in other cellular pathways including apoptosis, cell signalling and mineralisation ( Vance & Tasseva, 2013; Wu *et al*., 2008). Interestingly, despite complete loss of PSS1 in homozygous *Ptdss1* knockout mice, which resulted in a significant reduction in PS synthase activity, the mice appeared morphologically normal, viable and fertile ( Arikketh *et al*., 2008). Indeed, PS levels remained stable in most tissues, other than a modest reduction in the liver, presumably compensated for by PSS2 activity. In contrast, LMS was found to result from gain-of-function (GOF) mutations, leading to increased enzyme activity and blocking end-product inhibition of PSS1 ( Sousa *et al*., 2014). Several of the reported missense mutations are recurrent in unrelated patients, with clustering of other mutations in close proximity, indicating a highly specific role for these particular amino acids and/or their functional domains ( Piard *et al*., 2018; Sousa *et al*., 2014; Tamhankar *et al*., 2015).

The identification of genes that cause rare skeletal dysplasias and extreme bone mineral density phenotypes in humans, including osteosclerosis and craniotubular hyperostosis, along with analysis of vertebrate models of these conditions, has shed important insights into the mechanisms that regulate bone tissue homeostasis ( Goltzman, 2002). Several key cell types are known to regulate bone formation and homeostasis and are conserved in zebrafish ( Chatani *et al*., 2011; Dale & Topczewski, 2011; Eames *et al*., 2012; To *et al*., 2012). Chondrocytes are responsible for cartilage formation, which is composed primarily of collagen II/IX/XI, as well as proteoglycan and glycosaminoglycan, and serves as a scaffold within which osteoblasts and osteoclasts regulate bone mineralisation. Osteoblasts differentiate from mesenchymal stem cells through expression of factors that drive bone formation such as Runx2 and Osterix, through secretion of structural components of the bone matrix, enzymes and growth factors. The receptor ligand, RANKL, is expressed on the surface of osteoblasts, and binding of RANKL to its receptor, RANK, on the surface of monocytes stimulates their maturation into osteoclasts that resorb bone. This process is also negatively-regulated by the osteoblast-secreted decoy receptor, OPG, and so the RANK/RANKL/OPG signalling axis serves as a feedback mechanism to regulate bone turnover by osteoblasts and osteoclasts.

Human and zebrafish *PTDSS1* orthologues share 78% amino acid identity. We previously showed that microinjection of physiologically high doses of RNA encoding human mutant forms of *PTDSS1* found in LMS caused generalized embryo toxicity, including axial defects, eye loss and jaw cartilage patterning defects, whereas injection of wild-type RNA had no effect even at much higher doses ( Sousa *et al*., 2014). The frequency of these defects correlated with RNA dose, thereby serving as a biochemical readout of LMS gain-of-function mutation activity. The abnormal embryos in these experiments did not survive beyond the larval free-feeding stage, and microinjected RNA is relatively unstable in zebrafish embryos, lasting for typically less than 3 days. In contrast, mineralized bone only emerges after 6 days in the zebrafish jaw and 2 weeks in the skull, and the skeleton is only fully formed after ~10 weeks ( Eames *et al*., 2012; Mork & Crump, 2015; Schilling & Kimmel, 1997; Sharif *et al*., 2014). As such it is therefore not possible to investigate skeletal tissue development and homeostasis using transient RNA injections in zebrafish.

In this study, we used transpose-mediated transgenesis to stably express wild-type and mutant forms of human *PTDSS1* ubiquitously or specifically in chondrocytes, osteoblasts or osteoclasts in zebrafish. We report multiple genomic integration sites for each of 8 different transgenes, with variation in the number of integrations between individuals. Despite the presence of multiple integration sites, we failed to detect gene expression at either the RNA or protein levels. All transgenic lines, however, exhibited incompletely penetrant mild scoliosis of the vertebrae, which was never observed in non-transgenic clutch mates. Taken together, these results indicate that *PTDSS1* expression is either silenced to sub-detectable levels or inconsistent with formation of viable animals.

## Methods

### Zebrafish lines

Adult and embryonic zebrafish ( *Danio rerio*) embryos were obtained from a wild-type strain from the European Zebrafish Resource Centre and raised at 28.5°C in accordance with Home Office licence PPL 70/7892. ( Westerfield, 1993). Our study adopted the ARRIVE guidelines. In various assays, we generated experimental animals from an incross of heterozygotes, and wild-type and mutant animals were compared. Animals for comparison were matched for age and size. Analyses were performed blinded to genotype. All efforts were made to minimize any suffering of animals through environmental enrichment of their habitat, avoiding overcrowding, and monitoring animals for signs of distress or discomfort on a daily basis. *In situ* hybridization was performed using standard techniques with RNA probes labelled with digoxigenin (Roche) and detected using NBT/BCIP (Sigma).

### Plasmid construction

The *Tol2* kit, plasmid construction and an explanation of the recombination events was as previously published ( Kwan *et al*., 2007).

### Generation of entry clones

pDONRP4-P1r and pDONR221 were obtained as part of the Multisite Gateway Cloning kit (Invitrogen). The pDONR plasmids were maintained in *ccdB*-tolerant bacteria and grown in the presence of kanamycin and chloramphenicol. The WT human *PTDSS1* ORF was cloned into pcDNA3.1 (Addgene) and mutagenesis was performed to introduce the c.1058A>G (p.Q353R) mutation (QuickChange II Site-Directed Mutagenesis Kit, Agilent Technologies). These clones were amplified using the primers shown in Table 1. The resulting fragment was included with pDONR221 in a BP recombination reaction to generate WT and mutant middle entry clones. To generate the 5’ entry clones, different promotors were amplified from zebrafish cDNA using the primers given in Table 1. BP recombination with pDONRP4-P1r was performed. The 3’ entry clone p3E mCherry IRES was synthesised by Genscript (full sequence available from ( Seda *et al*., 2019)). Correct cloning was confirmed by Sanger sequencing using primers listed in Table 2.

**Table 1.** Primers used to construct *ptdss1* tol2 constructs.

Name	Description	Primers
**5′ entry clones** **(pDONRP4P1R *att*** **B4 *att*** **B1r)**		
p5Ebetaactin2	5.3 kb beta- actin promoter (ubiquitous)	F 5' *GGGGACAACTTTGTATAGAAAAGTTG*AATTCCAGTTTGAAGAAACTTTTCAAGA 3'
		R 5' *GGGGACTGCTTTTTTGTACAAACTTG*GGCTGAACTGTAAAAGAAAGGGAAACTG 3'
p5Ecol2a1a	1.87 kb collagen, type II, alpha 1a promoter (chondrocytes)	*F 5'GGGGACAACTTTGTATAGAAAAGTTG*CCTCTGACACCTGATGCCAATTGC 3'
		R 5' *GGGGACTGCTTTTTTGTACAAACTTG*TGCAGGTCCTAAGGGGTGAAAGTCG 3'
p5Erunx2	4 kb runt related transcription factor 2 promoter (osteoblasts)	F 5' *GGGGACAACTTTGTATAGAAAAGTTG*GGAATGGGACCTCATGTACCTTCG 3'
		R 5' *GGGGACTGCTTTTTTGTACAAACTTG*GGTCGCCACTTTCGCTCCCAAATT 3'
p5Ectsk	3.46 kb cathepsin K promoter (osteoclasts)	F 5' *GGGGACAACTTTGTATAGAAAAGTTG*CATATGGGGTAGGACTGTAAAAAGTC 3'
		R 5' *GGGGACTGCTTTTTTGTACAAACTTG*TCTGACCTGCAGTCAAAGGTGCAAA 3'
**Middle entry clones** **(pDONR221 *att*** **B1 *att*** **B2)**		
pDONR221ptdss1WT	1.23kb human ptdss1 ORF WT	F 5' *GGGGACAAGTTTGTACAAAAAAGCAGGCT*ATGGCGTCCTGCGTGGGGAGGCGGACCC 3'
pDONR221ptdss1mut	1.23kb human ptdss1 ORF Q353R	R 5' *GGGGACCACTTTGTACAAGAAAGCTGGGT*TCATTTCTTTCCAACGCCATTGGTGACT 3'

**Table 2.** Primers used for DNA sequencing of constructs.

Name	Primer 5' - 3'
tol2 exon 1 F	TCCCTTGCTATTACCAAACCAA
tol2 exon 1 R	TGGCTGCTTTTGGACTGTGC
tol2 exon 4 F	TCTGCTCACGTTTCCTGCTA
tol2 exon 4 R	ACAATCTAATGCCAGTACACGC
cmlc2 F	GTCCAGGTCGTTGGTTTCACTC
cmlc2 R	GGTCACTGTCTGCTTTGCTGTTGGT
GFP F	ATGGTGAGCAAGGGCGAGGAGCT
GFP R	CCCAGGATGTTGCCGTCCTCC
M13R2	GGAAACAGCTATGACCATGA
beta-actin tol2 R	ACCGGGAGGAAACCTACTTGAA
beta-actin tol2 F	TGAGAGAATGCAGAGGGACTTC
col2a1 tol2 R	CCCTGACTGTGTGCTCTGTA
col2a1 tol2 F	GTATTTCAGCGCTCAATGGGG
runx2 tol2 R	ATTATGCCACGGTCCACAGCTTC
runx2 tol2 F	CACTAGCGAGCTTGGCTCCATC
ctsk tol2 R	CTGTAGGTCTGTGCATATGTTGC
ctsk tol2 F	CATATCGAAACAATAGAAGTGCTCGG
ptdss1 tol2 R	TGGGGAAAGCTACACCACTGATG
ptdss1 seq F	GACATCCTGTTGTGCAATGG
ptdss1 seq R	CATGCCGTACAGACAGAGGA
ptdss1 tol2 F	GGACAAGATCTCTTCTCTAAGACC
mCherry tol2 R	CTAGGAATGCTCGTCAAGAAGAC
mCherry tol2 F	CAAACCACAACTAGAATGCAGTG
M13-20	GTAAAACGACGGCCAGTG

**Figure 1.**
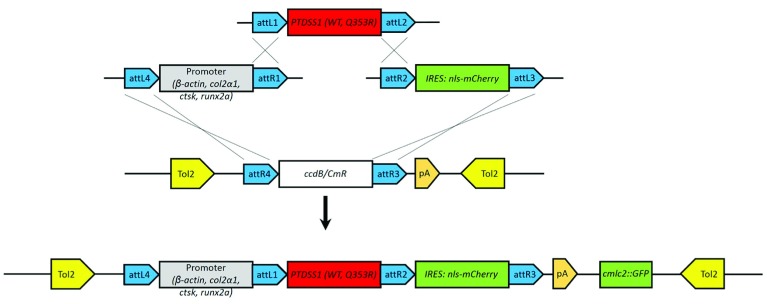
Cloning of tol2 constructs. Multisite Gateway© cloning was performed by combing a 5’ entry vector containing one of the four zebrafish promotors indicated, a middle entry vector encoding either wild-type (WT) or LMS mutant (Q353R) *PTDSS1*, and a 3’ entry vector encoding mCherry tagged with a nuclear localisation signal fused to a ribosome entry sequence (IRES), with a selectable Tol2 entry vector which also contained a separate *cmclc2:GFP* cistron for selection of animals with successful integration.

To generate plasmids for injection into zebrafish embryos, Gateway LR reactions were performed according to the manufacturer's recommendations (Invitrogen). LR II Plus clonase was used and the reaction performed overnight at 25°C. 3ul of each reaction was transformed into Top10 competent cells (Invitrogen) and grown on ampicillin plates. Clear colonies were picked for miniprep and screening ( Figure 1).

### Injections

On the morning of the injection, *Tol2* transposon DNA was mixed with an aliquot of *Tol2* mRNA at a concentration of 12.5 ng/µL of both DNA and mRNA, diluted with RNase-free water as required. The injection volume was calibrated to inject 1–2 nL of DNA:RNA injection mix. Embryos were injected at the one cell stage using Picospritzer III (Parker Hannifin). Injected embryos were transferred to Petri dishes and incubated at 28–30°C. At the end of injection day, any dead or unhealthy embryos were removed.

### Imaging

GFP and alizarin red images were captured using a Zeiss SteReo Lumar.V12 equipped with a Zeiss AxioCam HRc digital camera and Zeiss AxioVision Rel. 4.8 software.

### DNA/RNA extraction

To extract RNA and gDNA from the same zebrafish, the AllPrep® DNA/RNA Micro kit was used (Qiagen). Individual zebrafish (10 d.p.f) were lysed in 350ul buffer RLT plus using a micro tissue homogeniser and the protocol was followed. Genomic DNA was eluted in 50ul and RNA in 14ul.

### cDNA synthesis

The Moloney Murine Leukemia Virus Reverse Transcriptase (M-MLV RT) kit was followed (Promega) using 12ul of RNA. Samples were incubated for 90 minutes at 37°C followed by 80°C for 10 minutes.

### qRT PCR

qRT PCR was done using a T100 Thermal Cycler (BioRad) both on gDNA and cDNA for copy number and for gene expression analyses. DNA samples were diluted 1:10. Per sample 12.5ul SyBr Green (Applied Biosystems), 50ng each primer and 1.5ul water was added to 10ul of diluted DNA. Each sample was repeated in triplicate. The amplification parameters were: 50°C for 2 min, 95°C for 10 min, followed by 40 cycles of 95°C for 15 sec and 60°C for 1 min. An internal control, *EF1a*, was run for each sample tested. All the primers used are shown in Table 3.

**Table 3.** Primers used for RTq-PCR of transgene expression elements.

Name	Primer 5' - 3'
hu ptdss1 F	GAAAGGGACAAAAGGTTCTG
hu ptdss1 R	TTGGTGACTTTTGACTTGGA
GFP F	AAGGGCATCGACTTCAAGGA
GFP R	TGATGCCGTTCTTCTGCTTG
mCherry F	TCCCCTCAGTTCATGTACGG
mCherry R	GTCCTCGAAGTTCATCACGC
zEF1a F	CTGGAGGCCAGCTCAAACAT
zEF1a F	ATCAAGAAGAGTAGTACCGCTAGCATTAC

### Transient transfections

A 6 well plate of Hela cells was transfected with 1ug plasmid DNA using Fugene (Promega) following the manufacturers protocol. The cells were incubated at 37°C for 48 hours before harvesting.

### Western blotting

Cell pellets and zebrafish lysates were run on a western blot. Cell pellets were from a 6 well plate and zebrafish lysates contained 10 zebrafish from 3, 7 or 10 d.p.f. All samples were lysed in ice-cold NP-40 buffer (150 mM NaCl, 50 mM pH 8 Tris–HCl, 1% NP-40) containing 1× complete protease inhibitor cocktail (Roche) and 100 µM phenylmethanesulfonyl fluoride (Sigma). Insoluble contents were pelleted at 13 000 *g* for 30 min at 4°C and the supernatant was prepared in Laemmli sample buffer (Bio-Rad) containing 50 mM dithiothreitol. Samples were heated at ~90°C for 10 minutes before being run on a 10% polyacrylamide gel made in house, along with a marker (Biorad 1610376). Proteins were transferred to a membrane using the Trans-Blot® Turbo™ Blotting System (Biorad) and blocked overnight at 4°C in 5% milk, prepared in PBS/0.1%Tween 20 (Sigma) (PBST). Blots were incubated with the following primary antibodies for 3 hours at room temperature in blocking buffer: mCherry (1:2000), mouse (Anti-mCherry antibody [1C51], Abcam, ab125096) or p44/42 MAPK (Erk1/2) (1:400) rabbit (cell signalling, 9102S).

Blots were washed 4 x 5 minutes in PBST and incubated for 1 hour in PBST with the appropriate secondary antibody (1:5000). Blots were again washed 4 x 5 min in PBST and developed using Clarity Western ECL blotting substrate (BioRad) blots were visualised using the ChemiDoc imaging system (BioRad).

### Alizarin red staining of adult zebrafish

The protocol was performed at room temperature and each step was left overnight rolling. Adult zebrafish were fixed in 4% PFA/PBS followed by a rinse in tap water. The zebrafish were eviscerated and skinned, and then bleached in 1% KOH with 3% hydrogen peroxide. The following day the zebrafish were rinsed in tap water for 30 mins and subsequently 30ml saturated sodium tetraborate in 70ml water. The zebrafish were next stained with 1mg/ml alizarin red in 1% KOH. After 30 min rinse in tap water the zebrafish were cleared in 1% trypsin in 2% sodium tetraborate for several days. Once cleared the zebrafish were washed through a series of 1% KOH/100% glycerol solutions typically 20% final glycerol, 40% final glycerol and finally storage was in 70% final glycerol/30% alcohol (70%).

## Results

**Figure 2.**
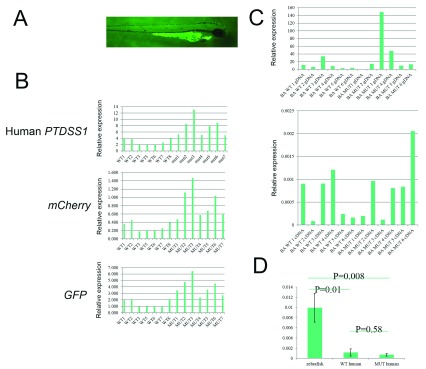
Expression analysis in transgenic zebrafish. **A**) Example of a zebrafish with a GFP positive heart. **B**) Copy number analysis of integrated human *PTDSS1*, *GFP* and *mCherry* by q-PCR on gDNA isolated from individual GFP- fluorescing zebrafish. **C**) Comparison of DNA copy number (top panel) and RTq-PCR expression analysis (bottom panel) for individual zebrafish where DNA and RNA were extracted from the same samples. **D**) Comparison of endogenous zebrafish *ptdss1* with human *PTDSS1* showing significantly higher levels of endogenous transcripts over WT and mutant transgene transcripts (p=0.01 and 0.008 respectively).

In total, 8 *Tol2* vectors were produced, consisting of either the zebrafish *beta-actin* (ubiquitous expression), *runx2* (osteoblast), *ctsk* (osteoclast) or *col2a1a* (chondrocyte) promoters fused upstream of the full length human *PTDSS1* ORF encoding either the wildtype sequence or the p.Q353R variant. We chose to study the p.Q353R mutation because it has been found to occur independently in multiple affected families ( Sousa *et al*., 2014). Since there is no existing information with which to predict whether tagged PTDSS1 is functional, we also included an mCherry cDNA sequence downstream of each promoter-PTDSS1 sequence separated by an internal ribosome entry site (IRES), which was itself downstream of the PTDSS1 stop codon to facilitate visualization of protein arising from this transgene. This entire cassette was expressed as a single cistron. In addition, the construct contained a GFP sequence driven by the heart specific promoter *cmlc2* in order to identify successful integration of the transgene by visual inspection of the heart ( Figure 1; Figure 2A). Each clone was Sanger sequenced to ensure sequence integrity.

**Figure 3.**
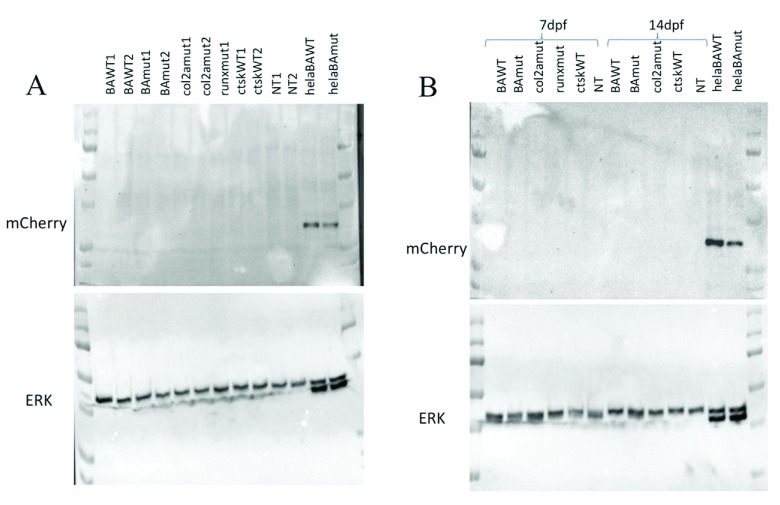
Analysis of transgene expression in transgenic zebrafish. A) Immunoblotting showing mCherry and ERK (control) in extracts from 3 d.p.f. transgenic zebrafish (either wild-type, Wt, or Q353R mutant, Mut) and non-transgenic (NT) zebrafish as well as HeLa cells transfected with the same beta-actin (BA) constructs. B) Similar western blot including transgenic zebrafish at 7 d.p.f and 14 d.p.f.

The beta-actin *Tol2* vectors were functionally tested by transient transfection into HeLa cells. Since antibodies reliably detecting PTDSS1 are not currently available, Western blotting using anti-mCherry antibody was therefore used to confirm expression from the β-actin promoter. Appropriate expression of mCherry was successfully detected in this way ( Figure 3) indicating that a functional promoter was operating and that the IRES successfully initiated translation of the mCherry protein.

All 8 *Tol2* vectors demonstrated successful integration with between 5.4% and 12.6% of embryos showing mosaic expression of GFP within the heart at 48 hours post-injection into one-cell stage zebrafish embryos. ( Figure 2A, Table 4). We subsequently bred these transgenic zebrafish beyond F5, which generated animals with completely green hearts, demonstrating stable integration of the transgene. Genomic DNA was extracted from individual 10 dpf β-actin transgenic zebrafish and tested for copy number of human *PTDSS1*, *GFP* and *mCherry* using qPCR. We confirmed the presence of all three regions of the transgene with relative copy numbers ranging significantly. Further there was a strong correlation between the relative copy number assessed using *PTDSS1*, *GFP* or *mCherry* primer sets in each embryo, confirming that the transgene had most likely integrated in its entirety at each site within the genome ( Figure 2B).

**Table 4.** Number of injections and % of zebrafish with GFP+ve hearts.

Transgenic line	Zebrafish injected	Green hearts	%
Beta-actin-PTDSS1-tol2 WT	1260	100	7.9
Beta-actin-PTDSS1-tol2 Mutants	1448	108	7.5
Col2a1a-PTDSS1-tol2 WT	1235	97	7.9
Col2a1a-PTDSS1-tol2 Mutants	1235	79	6.4
Ctsk-PTDSS1-tol2 WT	1420	77	5.4
Ctsk-PTDSS1-tol2-mutants	875	110	12.6
Runx2-PTDSS1-tol2 WT	700	65	9.3
Runx2-PTDSS1-tol2-mutants	600	55	9.2
Total	8773	691	7.9

Next, the expression of human *PTDSS1* zebrafish was determined by qRT-PCR using cDNA extracted from the same individual zebrafish on which the relative number of integrations had been analysed at the gDNA level. We did not detect expression of human *PTDSS1* in any of the 8 lines of transgenic zebrafish ( Figure 2C). In contrast, we did detect expression of the endogenous zebrafish *ptdss1a* gene ( Figure 2D). To further investigate transgene expression, whole mount *in situ* hybridization for human *PTDSS1*, *GFP* and *mCherry* was performed on 6 d.p.f beta-actin transgenic zebrafish and compared to non-transgenic controls. No specific expression of any of the probes was detectable (data not shown).

To investigate protein levels, protein lysates from transgenic zebrafish at different days post fertilization were analysed by western blotting. In each case, a negative control generated from non-transgenic zebrafish of the same age was used. The lysates from transient transfection of HeLa cells served as a positive control. No mCherry protein could be seen in any of the transgenic zebrafish tested, although it was clearly present in lysates from transfected HeLa cells ( Figure 3). In conclusion, while we robustly detected successful integration of the entire and apparently functional construct for each of the 8 transgenes in multiple lines of zebrafish, no gene product could be detected.

**Figure 4.**
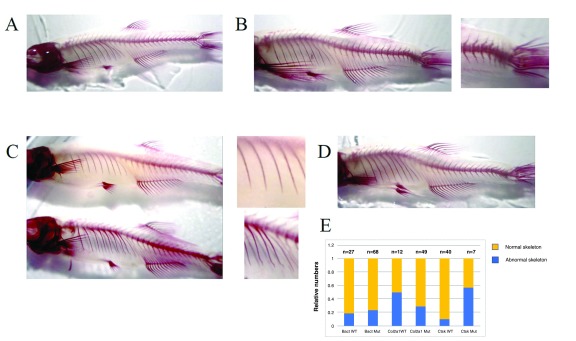
Imaging of zebrafish skeletons using alizarin red staining. Spinal cord, ribs and fin rays in **A**) a non-transgenic zebrafish; **B**) a transgenic zebrafish showing spine/tail kink; **C**) a transgenic zebrafish with scoliosis. **E**) Graphical representation of the distribution of skeletal defects seen for each transgenic line tested.

Because LMS features prominent skeletal features, we performed alizarin red staining on 6-month-old transgenic zebrafish to investigate skeletal mineralisation of transgenic zebrafish ( Figure 4A–D). We noted several prominent morphological defects that were never observed in non-transgenic animals of the same age, specifically sharp lateral bending of the caudal fin vertebrae and irregularities of the ribs, which were perfectly smooth in non-transgenic zebrafish ( Figure 4E). These defects were present in all transgenic lines of zebrafish, affecting between 10–60% of animals, with no obvious relationship to the particular mutation or the promoter used.

## Discussion

Zebrafish have generally proven to be a very useful tool for studying bone formation in vertebrates. Mineralization occurs in a predictable and stereotypic manner beginning at 3 dpf and craniofacial bones develop in a similar manner to those of higher vertebrates ( Eames *et al*., 2012; Mork & Crump, 2015; Mackay *et al*., 2013; Schilling & Kimmel, 1997; Sharif *et al*., 2014). Previous overexpression studies following injection of *PTDSS1* RNA showed no effects resulting from the wildtype sequence, while for the LMS mutant RNA, a marked effect on craniofacial structures such as widely spaced eyes, short jaw and a wide angle of Meckel’s cartilage was detected ( Sousa *et al*., 2014). A limitation to this methodology is the ability to study the transition from cartilage to mature bone and beyond. Therefore, in this study we set out to create a series of constitutive transgenic zebrafish carrying wildtype and mutant human PTDSS1 under the control of different cell lineage-specific promoters. This included the β-actin promoter, which would constitutively express the human *PTDSS1* in all transgene-containing cells and in a non-time dependant or differentiation-specific manner.

Overall, we were satisfied that intact plasmids had been cloned and transduced successfully. This was based on several lines of evidence starting with Sanger sequence validation of the integrity of each construct. Despite all of the promoters used in the Tol2 constructs being generated from zebrafish-specific sequences, it was possible to test the β-actin constructs following transfection in HeLa cells. This led to strong expression of mCherry protein as detected by western blotting ( Figure 3). This clearly indicated the presence of a functionally effective, zebrafish promoter sequence and the integrity of the IRES driving mCherry expression. Following transgenesis, we found a high percentage of the zebrafish injected with each construct to have GFP ^+^ hearts ( Figure 2A; Table 4), which showed that the *cmlc2-GFP* element of the construct integrated and was expressed. Furthermore, there was a high correlation for copy number of each different part of the transgene in individual zebrafish ( Figure 2B). This not only indicated that the construct integrated in multiple copies in some zebrafish but also that in each case, the transgenes were very likely to be intact.

We also performed several other experiments including qRT-PCR and western blotting of mCherry ( Figure 3). All methods failed to show any detectable RNA or protein expression of the transgene. Epifluorescence of mCherry, which we had hoped to serve as a live *in vivo* reporter of transgene expression, and also immunohistochemistry using an anti-mCherry antibody failed to produce a signal. For the latter we concentrated on mCherry detection since there are currently no good antibodies available for PTDSS1 and even if there were, data might have been compromised by cross-reaction with the endogenous zebrafish homologue. In contrast, mCherry transcripts and protein would be unique to the transgenic zebrafish and the available antibodies are well established, as demonstrated by the results obtained following HeLa cell transfection. Taken together, our data indicate that, in many zebrafish that we analysed, multiple integrations were successfully achieved, and that this resulted in limited transgene expression. We were able to demonstrate that the β-actin construct drove efficient gene and protein expression in transfection experiments using HeLa cells, which further shows that each independent element of the transgene is functional and suggests that this is not the explanation for failed transgene expression.

There are several possible explanations for the failed transgene expression. Formally, it is possible that the promoters are not active in zebrafish, however, this seems unlikely given that all four promoters have been shown to drive gene expression in zebrafish previously ( Chatani *et al*., 2011; Dale & Topczewski, 2011; To *et al*., 2012). Another possibility is that the transgenes are silenced which could relate to the site of transgene insertion within the 3D genome and chromatin conformation. However, we showed many zebrafish to carry multiple transgene insertions, and so this explanation would require multiple independent silencing functions to be active, which seems unlikely. It is also possible that elevated expression of *PTDSS1* is inconsistent with life, such that transgenic zebrafish expressing the transgene do not survive. It is unclear what the true explanation of these results is.

Previous studies have shown that PSS1 activity is normally under tight negative feedback regulation, while GOF mutations can attenuate this level of control ( Sousa *et al*., 2014; Vance, 2018). In contrast, simple over-expression of WT PSS1 in human hepatoma cells was shown to increase PS synthesis although the overall quantity of PS was not increased ( Stone & Vance, 1999). This was explained by two compensatory mechanisms, induction of PS decarboxylation and attenuation of PSS2 activity. Thus, PS homeostasis is maintained.

We also analysed adult zebrafish to see if any phenotype could be detected, particularly something attributable to mutant PTDSS1. In particular, we noted the variable occurrence of scoliosis, present in a proportion of the zebrafish. This was of particular interest since lumbar kyphoscoliosis was described in the LMS patient described by Majewski in 2000 ( Majewski, 2000), although not specifically recorded for any of the other patients reported. While scoliosis is one of the most common naturally occurring malformation in zebrafish ( Boswell & Ciruna, 2017), this usually occurs in the presence of pathogen infection and in very old zebrafish. By contrast, we never observe scoliosis in our aquatics facility at the ages reported here, and routine microbiological testing continues to exclude microbiological infections in our facility.

Our initial experimental design included wild-type and mutant PTDSS1 for each of the four promoters used, with the intention that the wild-type transgene would serve as a negative control. We also anticipated that PTDSS1 expression would have selective effects in only a subset of the skeletal cell lineages under study and would additionally affect the ubiquitous β-actin mutant transgenic zebrafish. Contrary to these hypotheses, we observed skeletal abnormalities in all 8 transgenic lines. There are several possible explanations for this. Firstly, accurate cellular dosing of PTDSS1 could be important in all three skeletal lineages that we investigated, and that this is reflected by both silencing of the transgene and the appearance of specific skeletal defects in all transgenic lines. While transient expression of wild-type PTDSS1 did not produce embryonic defects in our previous study ( Sousa *et al*., 2014), it is possible that long-term enhancement of PTDSS1 expression levels leads to cumulative defects with the bone. It is also possible that the bone malformations seen with alizarin red staining may be incidental findings, although we do note that similar defects were never seen in control non-transgenic animals. Whichever of these explanations is true, the incomplete penetrance of the phenotype and the lack of a matched transgenic control model makes continued investigation of these transgenic lines impractical.

In conclusion, although the collective evidence suggests that the zebrafish have successfully integrated the human *PTDSS1* WT or p.Q353R vectors respectively, we cannot visualize explicit RNA or protein expression or identify a specific phenotype in these zebrafish.

## Data availability

### Underlying data

Figshare: Analysis of transgenic zebrafish expressing the Lenz-Majewski syndrome gene PTDSS1 in skeletal cell lineages. https://doi.org/10.6084/m9.figshare.7732328.v3 ( Seda *et al*., 2019).

The project contains the following underlying data files:

- Figure 1– Figure 3
- Raw phenotype data
- Raw qPCR data
- P3E mCherry IRES (full sequence of the entry vector we used to cloning)

Data are available under the terms of the Creative Commons Zero "No rights reserved" data waiver (CC0 1.0 Public domain dedication).
