# Supplementary material for: Analysis of transgenic zebrafish expressing the Lenz-Majewski syndrome gene PTDSS1 in skeletal cell lineages
Source: F1000Res. 2019 Mar 11;8:273. [Version 1] doi: 10.12688/f1000research.17314.1 (PMC6557000; doi:10.12688/f1000research.17314.1)
Supplement: Peer review history

# Peer review: Reviewer response for version 1

**DOI:** 10.5256/f1000research.18933.r48264

**Published:** 2019-06-03

**Authors:** Carles Gaston-Massuet

**Affiliations:** 1 William Harvey Research Institute, Barts and the London School of Medicine and Dentistry, Queen Mary University of London, London, UK

The research in this article describes the use of zebrafish Tol-2 Kit transposase-system with Gateway constructs to express wild type and mutant form of the human PTDSS1 gene in zebra fish. The authors attempt correctly to generate a model Lenz-Majewski Syndrome previously shown to be caused by mutations in PTDSS1 gene. 

 The article is very well written and clear on the methods. The design of the constructs are well explained and correct, but the transgenic fish did not exhibit the predicted phenotypes. This is mainly due to the lack of expression of the transgene generated although integration of the transgene was achieved. The authors clearly explain several possibilities which could account for the lack of phenotype mainly as part of generating the transgenic fish. Although the results yield inconclusive results on the role of PTDSS1 in the Lenz-Majewski Syndrome in fish, I recommend the indexing of this article. I think that the lack of publications on technical challenges encountered in production of such transgenic lines will be useful for the zebra fish transgenic community that may encounter this type of difficulties in  performing such studies.

 Minor comments:

1. The Tol2-Kit is a transposon-mediated whilst the authors refer to it as a transpose-mediated (abstract and thereafter). Transposon-mediated would be more accurate way to refer to this system.
2. Figure 4 needs to be improved to make it easy to read for the none expert. Authors could add arrows to indicate the exact morphological abnormalities observed. Figure 4C: Which is the genotype of the transgenic fish, are both transgenics? I think the authors could improve the figure by adding more annotations and a clearer table of genotypes will help the general readability

I have read this submission. I believe that I have an appropriate level of expertise to confirm that it is of an acceptable scientific standard.

# Peer review: Reviewer response for version 1

**DOI:** 10.5256/f1000research.18933.r45567

**Published:** 2019-03-25

**Authors:** Jean-Marie Delalande

**Affiliations:** 1 Blizard Institute, Barts and The London School of Medicine and Dentistry, Centre for Immunology, Queen Mary University of London, London, UK

In this research article Seda *et al*., describes the use of zebrafish Tol-2 constructs to express wild type and mutant form of the human PTDSS1 gene. A mutation in this enzyme, causing gain of function, has been previously linked to Lenz-Majewski Syndrome (Sousa *et al.,*2014 ^1^). Despite adequate design the construct didn’t deliver the predicted outcome.

 It is clear from the material & methods, as well as control experiments, that the constructs were correctly designed and integrated. As show by figure 1 A&B. Tol-2-kit transgenesis is known to result in multiple insertions, which could have helped with this gain of function experiment, but also possibly could have driven the system beyond physiological limits and cause embryonic lethality. Using a bicistronic construct meant that the expression of the enzyme and the reporter gene were decoupled, which limited the usefulness of the reporter gene as indicator of transgene expression. As stated by the authors, a fusion protein might have resulted in the loss of enzymatic activity. However, the authors could mention this strategy in the discussion as some bifunctional enzyme-GFP fusions have been reported in the literature (Martin et al., 2009 ^2^). Using the Tol2-kit, clone 455 "pME-mCherry no stop" might have been worth exploring. The authors should also discuss the fact that several articles mention issues using bicistronic IRES constructs, as it is likely to be the main issue here. Hennecke *et al*., state; “However, failures of bicistronic applications have been observed, although these were often not published” (2001 ^3^). See also Mansha et al., 2012 ^4^. Considering the results in this study, it is possible that the constructs produced unstable PTDSS1 mRNA. Multiple insertions combined with transient mRNA background expression of the transgene might explain the scoliosis phenotype (which is more prevalent in the osteoblast ( *ctsk*) and chondroblast ( *col2a1a*) specific promoters I figure 4E.). 

 Overall because such results tend not to be published and issues with similar constructs might not have been previously reported. Therefore I recommend indexing of this article, as it will be useful to the scientific community in this field of research. 

 Minor points: 

- End of page 3: the methods part on *in situ *hybridization should be taken out as no *in situ*results are presented. 
- Figure 2C: results are a bit puzzling and could be discussed further. Is the relative expression just minimal background expression? It seems strange that it is negatively correlated with the number of inserted copies.
- Figure 4: picture could be presented in a table clearly stating which fish are wild type and mutant.

I have read this submission. I believe that I have an appropriate level of expertise to confirm that it is of an acceptable scientific standard.

## References

[1] SousaSBJenkinsDChanudetETassevaGIshidaMAndersonGDockerJRytenMSaJSaraivaJMBarnicoatAScottRCalderAWattanasirichaigoonDChrzanowskaKSimandlováMVan MaldergemLStanierPBealesPLVanceJEMooreGE: Gain-of-function mutations in the phosphatidylserine synthase 1 (PTDSS1) gene cause Lenz-Majewski syndrome. *Nat Genet*.2014;46(1) : 10.1038/ng.2829 70-6 10.1038/ng.2829 24241535

[2] MartinLCheAEndyD: Gemini, a bifunctional enzymatic and fluorescent reporter of gene expression. *PLoS One*.2009;4(11) : 10.1371/journal.pone.0007569 e7569 10.1371/journal.pone.0007569 19888458PMC2766624

[3] HenneckeMKwissaMMetzgerKOumardAKrögerASchirmbeckRReimannJHauserH: Composition and arrangement of genes define the strength of IRES-driven translation in bicistronic mRNAs. *Nucleic Acids Res*.2001;29(16) :3327-34 1150487010.1093/nar/29.16.3327PMC55851

[4] ManshaMWasimMPlonerCHussainALatifAATariqMKoflerA: Problems encountered in bicistronic IRES-GFP expression vectors employed in functional analyses of GC-induced genes. *Mol Biol Rep*.2012;39(12) : 10.1007/s11033-012-1898-z 10227-34 10.1007/s11033-012-1898-z 23076521
